# Prolonged CT urography in duplex kidney

**DOI:** 10.1186/s12894-016-0139-5

**Published:** 2016-05-13

**Authors:** Honghan Gong, Lei Gao, Xi-Jian Dai, Fuqing Zhou, Ning Zhang, Xianjun Zeng, Jian Jiang, Laichang He

**Affiliations:** Department of Radiology, the First Affiliated Hospital of Nanchang University, 17 Yongwai Zheng Street, Donghu District, Nanchang, Jiangxi 330006 China

**Keywords:** Duplex kidney, Duplicated ureters, Prolonged-delay contrast enhancement, Multi-slice spiral CT urography

## Abstract

**Background:**

Duplex kidney is a common anomaly that is frequently associated with multiple complications. Typical computed tomography urography (CTU) includes four phases (unenhanced, arterial, parenchymal and excretory) and has been suggested to considerably aid in the duplex kidney diagnosi. Unfortunately, regarding duplex kidney with prolonged dilatation, the affected parenchyma and tortuous ureters demonstrate a lack of or delayed excretory opacification. We used prolonged-delay CTU, which consists of another prolonged-delay phase (1- to 72-h delay; mean delay: 24 h) to opacify the duplicated ureters and affected parenchyma.

**Methods:**

Seventeen patients (9 males and 8 females; age range: 2.5–56 y; mean age: 40.4 y) with duplex kidney were included in this study. Unenhanced scans did not find typical characteristics of duplex kidney, except for irregular perirenal morphology. Duplex kidney could not be confirmed on typical four-phase CTU, whereas it could be easily diagnosed in axial and CT-3D reconstruction using prolonged CTU (prolonged-delay phase).

**Results:**

Between January 2005 and October 2010, in this review board-approved study (with waived informed consent), 17 patients (9 males and 8 females; age range: 2.5 ~ 56 y; mean age: 40.4 y) with suspicious duplex kidney underwent prolonged CTU to opacify the duplicated ureters and confirm the diagnosis.

**Conclusion:**

Our results suggest the validity of prolonged CTU to aid in the evaluation of the function of the affected parenchyma and in the demonstration of urinary tract malformations.

## Background

Duplex kidney is a common anomaly of the urinary system [1]. Most patients are asymptomatic, with this anomaly being detected incidentally on imaging studies performed for other reasons. Symptomatic patients usually have complete ureteric duplication in which the ureters are prone to developing obstruction, reflux, and infection. Despite being easily detected on excretory urography, ultrasonography, computed tomography (CT) and magnetic resonance imaging (MRI), the affected parenchymal function and anatomic variations of duplex kidney are frequently difficult to assess using these imaging modalities [2]. Assessment of the affected parenchymal function and visualization of the entire duplicated ureters/collecting systems are particularly important for surgical planning and long-term follow-up.

Croitoru et al. [3] have previously reported percutaneous injection of iodinated contrast medium into the dilated collecting systems, and intravenous contrast administration was performed. The resultant 3D images clearly showed the entire ectopic ureter in various planes. This method is very useful when evaluating duplicated ureter-related abnormities. However, this method cannot reveal the function of the affected duplex kidney, and the method is also invasive.

In the current study, we first presented prolonged-delay CT urography (CTU), which is a promising protocol to evaluate renal resorption and outline anatomic variations and urinary tract obstruction. We next summarized and discussed the prolonged findings of 17 patients with duplex kidney at our hospital since 2001.

## Methods

### Subjects

The institutional review board of the First Affiliated Hospital of Nanchang University approved this retrospective study and waived the requirement for patient informed consent. Between January 2005 and October 2010, seventeen patients (9 males and 8 females; age range: 2.5 ~ 56 y; mean age: 40.4 y) with duplex kidney were included in this study. These patients demonstrated a renal mass on sonography, MRI or CT. Two blinded abdominal radiologists (Honghan Gong with 40 years of experiences and one of three others, each with up to five years of experiences) independently reviewed the images for the systemic evaluation and diagnosis. Of the seventeen patients with duplex kidney in our study, 15 presented with urinary tract infection and 2 with ectopic ureteral orifice for hospital visits.

### CT

Prolonged-delay CTU images were obtained from two CT scanners: Somatom Sensation 16 (Siemens Medical Solutions, Forchheim, Germany) and Aquilion 64 (Toshiba Medical Systems, Tokyo, Japan). The CT scanner calibration was checked weekly and adjusted if needed. CTU images were obtained using varying tube current (150–180 mA from the scanner depending on the patient size) and 120 ~ 140 kVp. The scans were performed with a pitch of 1.0–1.4, a rotation time of 0.5 to 0.8 s, a section thickness of 5–10 mm, a table speed of 7–10 mm/s, and a standard reconstruction algorithm. The target area was from the top of the kidneys to pubic symphysis for each phase. Oral hydration was used for the children prior to scanning.

We first performed unenhanced scanning, and then CTU after a 20-s delay from the beginning of the single-bolus administration of the intravenous contrast agent. Each patient received 90–100 ml of a contrast agent with 300 mg of iodine per milliliter (iopromide 76 %; Iopromide 300; Bayer Schering Pharma AG) at a rate of 3.5–4 ml/s using a power injector (MEORAO-Stellant; MEORAO Company, Germany). CTU included four-phase (unenhanced, arterial, parenchymal and excretory) scanning. After CTU, the radiologist checked each phase immediately to decide whether a prolonged-delayed phase was needed.

Depending on the excretory opacification of duplex kidney, prolonged CTU scanning was performed 1–72 h (mean duration: 24 h) after typical CTU. Each patient received 2–4 (mean: 3 times) prolonged-delay scans using the same scanning parameters as those for typical CTU. Delayed imaging was restricted to the abdomen and was performed about one hour after the initial administration of the bolus using the same imaging parameters. Prescan scout CT images were obtained prior to prolonged-delayed scanning to ensure complete contrast opacification of the duplicated ureters.

### Image and data analyses

Multi-planar reconstruction (MPR), volume rendering (VR), maximum intensity projection (MIP), curved-planar reconstruction (CPR) and blood vessel probing were performed using a work station (vitrea@2 version 3.7.0).

## Results

The demographic characteristics are summarized in Table 1. Among the 17 confirmed duplex kidneys, 13 were unilateral duplex kidneys, and four were bilateral duplex kidneys; 11 cases were confirmed surgically, and six cases were confirmed at the follow-up.

**Table 1:** Demographic characteristics of 17 patients with duplex kidney

Patient demographics	
Age	2.5 ~ 56y, mean 40.4y
Sex	9 male/8 female
Category	13 unilateral/4 bilateral
Irregular small kidney/Megaureter malformation Ectopic ureterocele	7
Renal pelvis/Ureter malformations	5
Bladder-ureter malformation	5

**Fig. 1:**
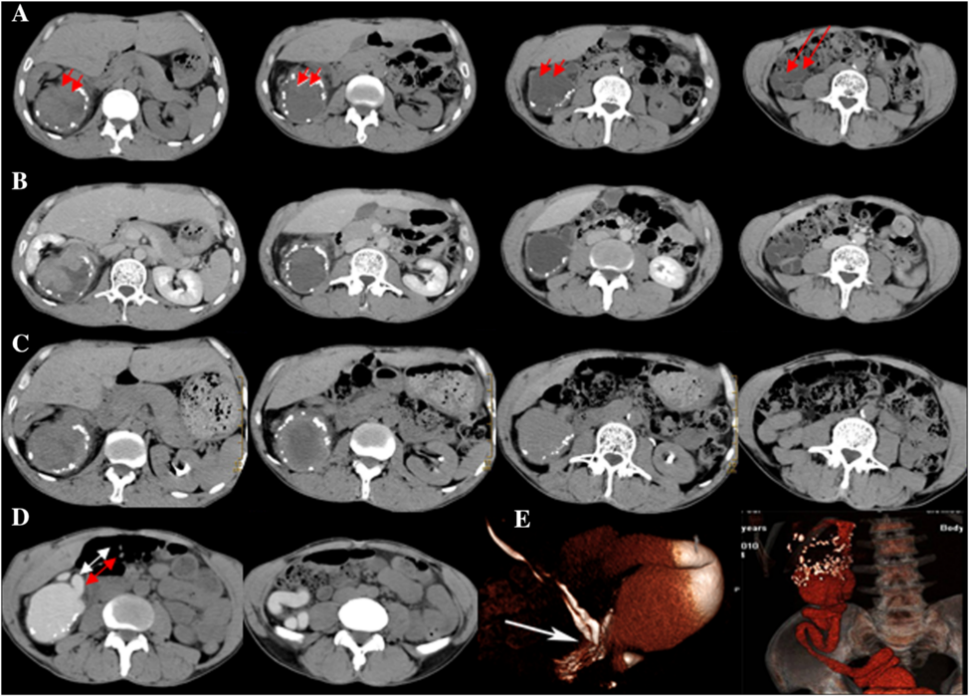
Case 1. A 56-year-old male patient with right side duplex kidney. Unenhanced supine axial CT (**a**) shows a solitary round, iso-dense, soft-tissue mass with a clear boundary (*short arrow*). The irregularly annular calcified shadow around the mass was equivalent to multiple annular low-density shadows in the ileocecal junction (*long arrow*). The mass was not clearly intensified after contrast enhancement (**b**) and one-hour delayed (**c**) CTU scanning. Interestingly, it was obviously strengthened after 18-h delay (**d**), confirming duplex kidney. Furthermore, we found a band of high-density shadows that was confirmed to be duplicated ureters (*double-headed arrow*). Reformatted 3D CTU after segmentation of bone structures also showed the entire course of the dilated ureters (**e**)

**Fig. 2:**
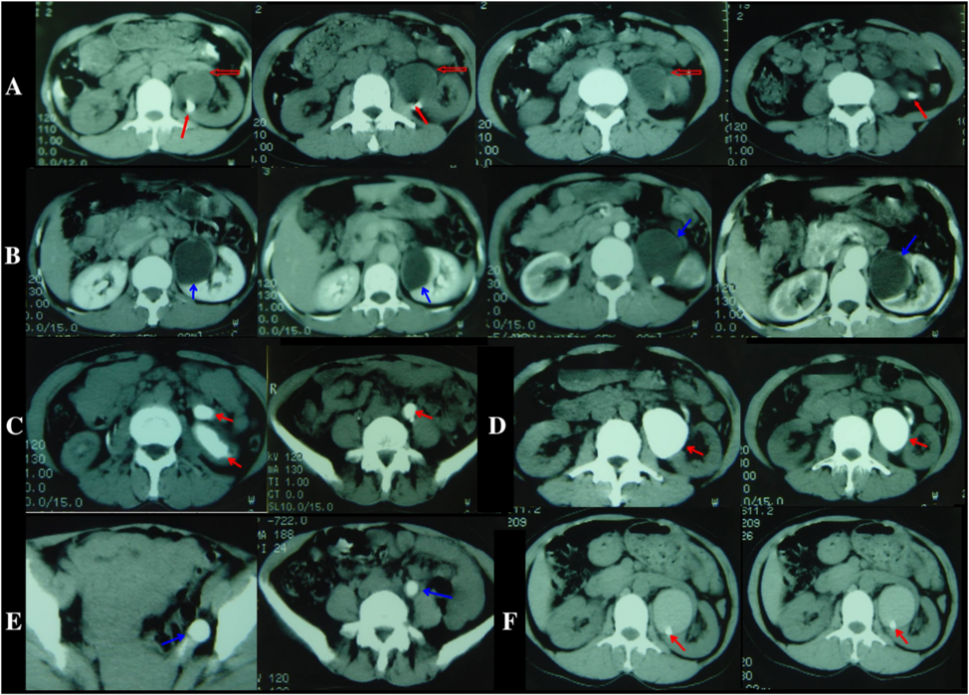
Case 2. CT scans obtained in a 46-year-old woman with left duplex kidney & ectopic ureter openings. Non- enhanced scan obtained at the level of the upper-middle pole of the left kidney shows areas of abnormal low-density (*red faint arrow*) and stone shadow (*red solid arrow*) (**a**). Contrast-enhanced scans obtained at the same level as in (**a**). The upper-middle pole of the left kidney (blue arrow) shows no enhancement on the 6-min scan (**b**) and abnormal low density enhancement on the 3 h (**c**), 18 h (**d**), 24 h (**e**) and 45 h (**f**) scan, and ectopic ureter openings can be seen in (**e**) (blue arrow)

**Fig. 3:**
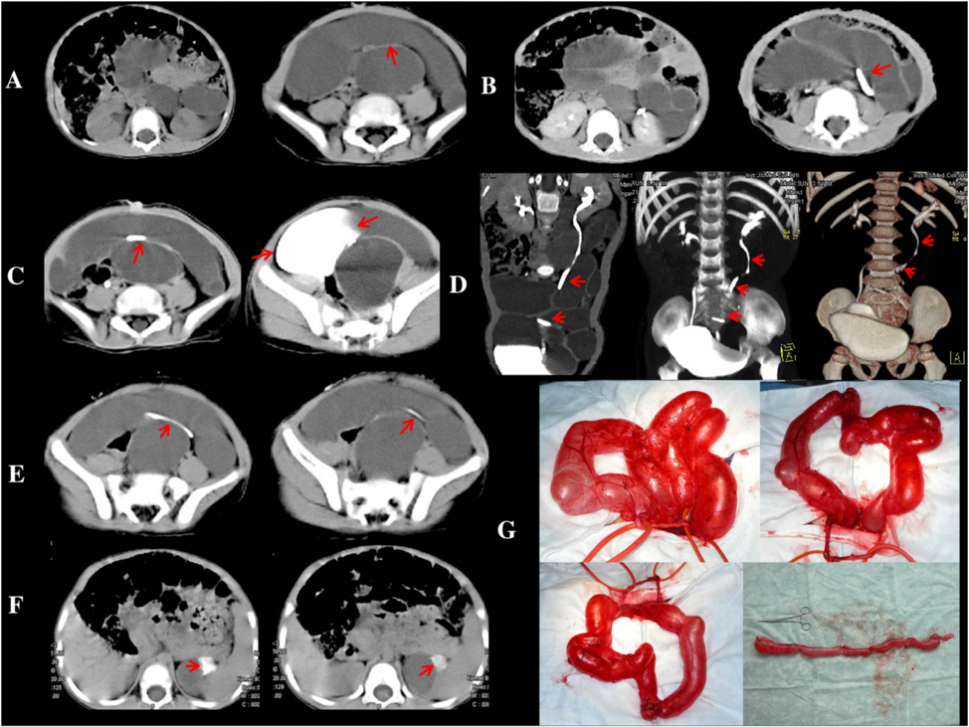
Case 3. CT obtained in a 3.5-year-old boy with left duplex kidney & ureters, and congenital megaflop-ureter. Non- enhanced scan obtained at the abdomen & pelvis shows fluid-filled loops, the normal bowel loops are compressed to the right side of the abdominal wall, and a tendon-like shadow can be seen in the bowel loop-like areas (red arrow) (**a**). The tendon-like shadow was intensified as the normal ureter (red arrow) (**b**), and the normal bladder was also intensified in the left side pelvis (*red arrow*) on the 54-min scan (**c**). Meanwhile, MIP reconstruction shows the position and course of left ureter changed (*red arrow*) (**d**). The left ureter shows delayed enhancement on the 3-h and 13-min scan (**e**). The area of the left side kidney shows a little irregular enhancement on the 23 h and 9-min scan (*red arrow*) (**f**). Operation findings (**g**)

**Fig. 4:**
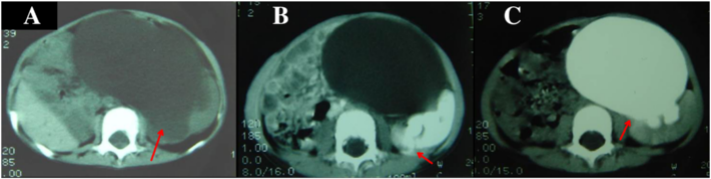
Case 4. CT scans obtained in a 2.5-year-old girl with left duplex ureters and congenital megalo-ureter. Non-enhanced scan obtained at the level of the inferior-middle pole of the left kidney shows a huge round-like area of low density (*red arrow*) (**a**). Contrast-enhanced scans were obtained at the same level as in (**a**), this area showed no enhancement on (**b**) the 26-h scan, and the tortuous contrast agent can be seen retroperitoneally (*red arrow*) (**b**). The huge round-like area of low density was intensified on the 48-h scan (*red arrow*) (**c**)

**Fig. 5:**
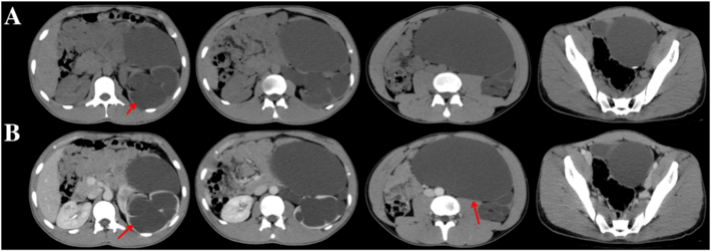
Case 5. CT scans obtained in a 20-year-old man with left congenital megaloureter. Nonenhanced scan obtained at the abdomen & pelvis shows huge fluid-filled loops (*red arrow*), and the normal bowel loops are compressed to the right side of the abdominal wall (**a**). The left renal pelvis and calyces show marked dilation, with parenchyma thinning (*red arrow*) on parenchymal phase enhancement (**b**), for unknown reason the images of prolonged phase were missing

Unenhanced scanning did not find typical characteristics of duplex kidney, except for irregular perirenal morphology. Duplex kidney could not be confirmed on typical four-phase CTU, whereas duplex kidney was easily diagnosed in axial and 3D reconstruction on prolonged-delay CTU (prolonged delay phase). All 17 cases had irregular perirenal morphology, 7/17 had irregular small kidney-Megaureter malformation or ectopic ureterocele, renal pelvis or ureter malformations, and 5/17 had bladder-ureter malformation (Figs. 1-5). Renal function of duplex kidney could be well assessed in prolonged-delay CTU (Figs. 1-5).

## Discussion

Ureteral duplication is often associated with vesicoureteral reflux, ureterocele, or ectopic ureter opening [4]. In most cases, the duplex kidney is divided into two parts—the upper and inferior poles—each with its separate collecting systems. In some cases, however, the kidney is entirely separated into two independent parts, each with its own renal pelvis and ureter. The diagnosis of duplicated ureters was previously based on excretory urography and retrograde ureteropyelography, and now has been replaced by CTU. Ultrasonography, CT and MRI have certain value in the evaluation of duplex kidney [5]. Ultrasonography can display the nonfunctioning upper pole and would be useful to differentiate renal cyst from renal pelvic diverticulum. CTU and MRU can delineate essentially all abnormalities of the collecting systems, ectopic ureter, and ureterocele that benefit the diagnosis of duplicated and ectopic ureter [6–8]. However, these protocols are mainly useful in evaluating duplex kidney with approximately normal function and simple anomaly [5]. However, in some cases, because of poor function and obstruction, a lack of or delayed excretory opacification limits the complete diagnostic features. Duplex kidney can be misdiagnosed as a renal or perirenal neoplasm, a renal cyst, a kidney tumor, or, occasionally, adrenal gland neoplasms. When presented with various complicated complications, duplicated ureters can be misdiagnosed as a cyst, an ectopic ureter, a ureterocele or ureteral obstruction. The opening of duplicated ureters may be outside the bladder or even extracorporal. It is difficult to show an ectopic opening from cystoscopy. Additionally, ultrasonography, CT or MRU can not effectively display the tortuous ureter and ectopic ureter openings, particularly in duplex kidney with prolonged dilatation.

CTU has basically replaced excretory urography for its advantages in anatomical details, fast imaging, high-quality 3D reconstruction and safety. The CT Urography Working Group of the European Society of Urogenital Radiology (ESUR) has proposed a clear definition of CTU in their Society Meeting in 2006 [9]. An issue was raised concerning the number of phases, including four-phase CTU (unenhanced, arterial, parenchymal and excretory), three-phase CTU (unenhanced, nephrographic and excretory), and two-phase CTU (unenhanced and combined nephroexcretory). In the present study, we reviewed a group of 17 patients who had duplex kidney malformations and various ureteral abnormal complications with enhanced/delay CTU (see Figs. 1-5). Prolonged CTU must include: (a) unenhanced axial scan; (b) arterial enhancement phase; (c) parenchymal phase; (d) excretory phase and a (e) prolonged-delay enhanced excretory period. Thus, the full course of prolonged-delay CTU includes five phases, differing from the ESUR-recommended or Croitoru et al. protocol reported previously [3]. (1) Our prolonged CTU adopted a single-bolus administration rather than two bolus injections of 300 mg/kg. (2) The delayed duration should be 72 h as the longest and 1 h as the shortest, not just several minutes of delay; in our experience, satisfactory opacification can be achieved 24 h after CTU in most cases. (3) Confine the X-ray beam to the target area to reduce the exposure of X-ray. For the duplex kidney with normal renal function, the ESUR-recommended CTU is sufficient to evaluate the malformations and related complications; in our case, the scanning can be terminated in the excretory phase. The main significance of prolonged-delay enhanced CTU is that there is sufficient delay time for duplex kidney with poor function to secrete contrast to the duplicated ureters; however, other considerations include the following: (1) confirming the duplex kidney malformation near the normal kidney; (2) exposing the entire course of the duplicated ureters and existing complications; (3) extending the period helps reflect the parenchymal function of duplex kidney.

Ectopic ureter is likely to occur with dysfunction, making it difficult to detect the ureter in complete duplex kidney with duplicated ureters with poor function. 3D reconstruction is a good method in delayed excretory urography, and a large amount of axial plane data has been condensed to the coronal plane to better display the dilated collecting systems.

Our preliminary results indicate that duplex-collecting system abnormalities can be successfully displayed by prolonged CTU. Given that prolonged-delay enhanced CTU is sensitive to duplex kidney with poor function, 3D reconstruction can clearly display the entire course of the ureter in multi-planes. Our results suggest that prolonged CTU has more advantages over other imaging modalities. Despite the relatively small sample in the current study, we have successfully examined the 17 cases of duplex kidney and its complications. We believe that prolonged CTU is a better choice in evaluating duplex kidney with poor function.

Beyond the benefits of prolonged CTU, a controversy focuses on radiation exposure. Are the radiation and time worthwhile, particularly in children? This is really an issue that must be addressed. Indeed, this practice results in higher radiation exposure and longer time involvement. However, we believe the benefits from this practice outweigh the risks. First, prolonged scanning provides a straightforward avenue to achieve greater, if not maximal, ureteral opacification, avoids detours and improves efficient diagnosis. Second, delayed CTU may be the only way to outline the entire course of the ureters, and, indirectly, index function in the affected parenchyma in the duplex part of the kidney, a feature that other imaging modalities cannot match. Third, a definitive diagnosis is particularly important for surgical planning and long-term follow-up; this advantage is much greater than the radiation risk.

There are several limitations in this study. First, the delayed phases were obtained in a wide spectrum of time intervals (1–72 h); in our experience, satisfactory opacification in this protocol can be achieved 24 h after CTU in most cases; however, in some cases, satisfactory opacification can be faster (1 h) or slower (72 h). Second, this study is retrospectively summarized over a 5-year period on 2 different scanners and a small sample size; larger samples are needed to refine our conclusion.

## Conclusions

Our results suggest the validity that prolonged CT urography could aid in the evaluation of the function of the affected parenchyma and in the demonstration of urinary tract malformations.

## Ethics approval and consent to participate

The institutional review board of the First Affiliated Hospital of Nanchang University approved this retrospective study and waived the requirement for patient informed consent.

## Consent for publication

Not applicable.

## Availability of data and materials

Not applicable.
